# Reflections on Reflection: Clarifying and Promoting Use in Experienced Coaches

**DOI:** 10.3389/fpsyg.2022.867720

**Published:** 2022-05-04

**Authors:** Christine Nash, Alan C. MacPherson, Dave Collins

**Affiliations:** ^1^Institute for Sport, PE and Health Sciences, University of Edinburgh, Edinburgh, United Kingdom; ^2^Grey Matters Performance Ltd., Stratford-upon-Avon, United Kingdom

**Keywords:** reflective practice, coaching, stimulated recall, expertise, knowledge criteria, challenge

## Abstract

**Background:**

We draw on the work of established scholars in the field of reflective practice who highlight its importance as a key cognitive skill for professionals to hold. While the substantive effect of engaging in reflective practice is emphasised in the literature, apparently coaches only spend a limited time learning about and engaging with it.

**Objectives:**

This study was conducted in two parts: Part 1 examined coaches’ knowledge of reflective practice and ascertained their perceived lack of value and use of reflective practice within their coaching. In response to the unexpected findings in Part 1, in Part 2, we instituted an educational intervention to further these participant coaches’ knowledge of Reflective Practice (RP) and facilitate its integration into their coaching practice.

**Design:**

The present study utilised a mixed method design with semi-structured interviews being conducted in Part 1. A coach development reflective programme inspired by Stimulated Recall approach was implemented in Part 2.

**Participants:**

Twelve high level coaches were interviewed about their reflective practices in Part 1. In Part 2, the same coaches agreed to participate in the educational intervention for the duration of the project.

**Results:**

Findings from Part 1 revealed an interesting paradox: coaches demonstrated a lack of appreciation for reflective practice yet recounted the positive influence that specific events and individuals had on their practice. In Part 2, to fully develop RP with the present cohort, an educational intervention was conducted. While watching videos of their own practice, coaches initially required lots of prompts from the lead interviewer to facilitate a deep and meaningful discussion of their practice. During the latter stages of the intervention, however, participants were less dependent on questions and prompts.

**Conclusion:**

In part 1, the coaches in this study did employ reflection, although they did not label it as such. The qualitative evidence we have gathered enables us to suggest that it is the combination of how to reflect, and against what criteria that makes RP a powerful tool to develop expertise which it has the potential to be. Importantly, however, additional coach education input is necessary for these benefits to be fully realised.

## Introduction

The importance of reflecting on cognition and action as part of the learning process has been emphasised by many investigators (Dewey, 1933; Schön, 1983; Argyris, 1993; Eraut, 1994). Accordingly, reflective practice has come to be recognised as a core element of professional expertise, becoming particularly prominent in the education (Jung, 2012), healthcare (Ghaye and Lillyman, 2010), and specific social policy professions (Tambornino, 2007). The term Reflective Practice (RP) refers to the ability to analyse one’s own practice, the incorporation of problem solving into learning by doing, and the application of critical theory to the examination and enhancement of professional practice. The terms reflective practice and reflexive practice are often used interchangeably: reflexive practice involves examining judgements, practices, and belief systems (Braun and Clarke, 2019), whereas RP is indicative of meaningful learning, development, and change for the individual and is situated in one’s professional context (Nash, 2021). Therefore, RP incorporates and builds upon the notion of “reflexivity” as an important, constituent component.

The evolution of RP has drawn on a variety of research perspectives: philosophy and scholarship of Dewey (1933) on this topic have influenced the development of several theories as to how individuals construct knowledge through experience (cit. Kolb, 1984, experiential learning theory; Schön, 1983, reflective practice theory). Importantly, there is a common thread to these theories, which states that knowledge and learning must be fundamentally embedded in the activity and its context. Consequently, these theories posit that the development of knowledge is dependent on reflecting on significant moments encountered in the activity. Therefore, in coaching, as one of many professional settings that Schön’s theory pertains to, it is proposed that the construction of domain-specific knowledge occurs when knowledge schemas are enriched by the conscious engagement with and reflection on experience. Similarly, within education, Dewey (1897, p. 79) conceived of ‘a continuing reconstruction of experience’ that translates seamlessly, in our opinion, into the applied environments of sport coaching (*cf*. Day and Newton, 2016). Schön coined the phrase ‘the reflective practitioner’ which is now used in many educational environments. Crucially, according to Schön, the ‘reflective practitioner’ is able to interrogate experiences and is therefore well equipped to deal with the messy realities of coaching practice (*cf*. Nash, 2016).

The complex relationship between knowledge, expertise, and experience that make up these realities is one that has raised many questions. For example, what is the relationship between expertise and experience (Persky and Robinson, 2017) and how does the conceptualisation of expertise relate to knowledge (Grundmann, 2017)? To add to this messiness, in several situations, the words knowledge, expertise, and experience are used interchangeably but often, as a consequence, inaccurately (Nash, 2021). While we acknowledge the importance of teasing apart operational definitions, we are curious to determine, in light of strong theoretical evidence if RP can be used to develop bespoke solutions to address just such messy, novel problems.

Reflecting these potential benefits, recent literature has established the importance of RP in ongoing professional development (Murdoch-Eaton and Sandars, 2014; Tracey et al., 2014; Körkkö et al., 2016), emphasising the benefits of engaging in a continual cycle of reflection, learning, and acting. Schön (1987) developed this argument further by claiming that RP is one of the cornerstones of a profession. This is a key concept in the present study: RP is emphasised in the nascent coaching literature, but there is doubt among coach educators and developers as to the amount and quality of RP coaches currently undertake, due in part to the curriculum design of coach education programmes. Therefore, discovering and implementing the means to learn RP ‘on-the-job’ are a useful skill for coaches (and coach educators) at all levels. Although coaching is not currently viewed as a profession in the traditional sense, there are attempts to address both its initial education provision and ongoing development. Consequently, and given the literature reviewed thus far, the widespread employment of RP is likely to play an integral role in this process. Indeed, RP is one of the key factors of developing expertise in coaching (Mamede and Schmidt, 2005; Morgan et al., 2020).

Therefore, given its potential importance, it is crucial to reflect on how RP can best be developed. Although it has been demonstrated that attendance at coach education courses increases the knowledge base of the coach, research conducted by Abraham and Collins (1998) demonstrates that the effectiveness of the coach may often remain unaltered. Implicitly, this finding suggests that coach education, if it is to realise its goals, requires prolonged and intensive engagement, with RP at its core. The social element of coach development has also been under-exploited (*cf*. Stoszkowski and Collins, 2014). Consequently, while knowledge in its various forms is of undoubted value, aspiring coaches need to make contacts and network within coaching environments, set goals for their development, gain applied experience, and reflect upon practice in its totality. Furthermore, it must be acknowledged that coaches, as mature learners, identify principles in the learning process that are key to effective engagement. According to Merriam (2001), these are involvement in their learning and evaluation of the results, most likely utilising a problem-solving approach. However, as adults tend to have less time to invest, they develop principles of andragogy, where learning is centred on tasks that directly relate to their job, in this case coaching (Knowles, 1984). In this vein, Mezirow (1990) argued that for effective adult learning to enable and drive reflection, it was vital that meaning schemas be explored and uncovered to enable a greater understanding of the values, beliefs, and decision-making practices that underpin the coaching process—by coaches themselves. Moreover, Mezirow goes on to state that this process of ‘reflecting back on prior learning to determine whether what we have learned is justified under present circumstances. This is a crucial learning process egregiously ignored by learning theorists’ (p. 5). Given the positive aspects outlined here, the concept of cognitive apprenticeships proposed by Collins and colleagues (*cf*. Collins et al., 1991) are apposite and timely. However, given the fractured state of coach education and development and the disagreements that persist among this community (Pill et al., 2021), these benefits may require to be considered more fully.

Current research evidence suggests that RP is considered beneficial to professional practice (Attard and Armour, 2006; Fenoglio and Taylor, 2014; Bosangit and Demangeot, 2016); however, given our collective experiences working with coaches in formal and informal settings, we had a tacit belief that coaches in and around our professional networks were not proponents of RP. Therefore, we wanted to examine how experienced and knowledgeable coaches perceive RP: this is the main aim that is enacted in Part 1 of the present paper. As a result of our unexpected findings in Part 1, in Part 2, we instituted an educational programme to explore and potentially enhance these participant coaches’ knowledge and integration of reflection into their coaching practice. The programme included three online RP workshops and a stimulated recall approach. We have presented findings as Part 1: initial experiences and perceptions of RP and Part 2: educating about/changing perceptions of RP.

## Part 1: Initial Experiences and Perceptions of RP

Building from the strong support for RP in the literature, the aim for Part 1 was to investigate high level coaches’ understanding of their learning journeys, their knowledge of reflection and how this had been facilitated. To understand how these coaches process experiences in their professional lives, in Part 1, a thematic analysis is employed (Braun and Clarke, 2006), and in so doing, it enables us to formulate a detailed account of this complex area.

### Methods

#### Participants

Participants included 12 high level coaches, from various sports (see Table 1; *M*_age_ = 44.58, *SD* = 4.27). Each participant was recruited by personal contact (Thomas, 2006) to take part in one-to-one interviews. All had been coaching at international level for at least 10 years (*M* = 15.75, *SD* = 3.60). Prior to the study, all participants stated that they were familiar with the notion of reflection but did not incorporate it into their coaching, nor did it constitute part of their ongoing professional development. Institutional ethical approval was secured prior to data collection commencing and informed consent was obtained from each participant.

**Table 1:** Participant coach details.

Coach	Sex	Sport	Age	Experience
1	Male	Tennis	41	13
2	Female	Swimming	37	12
3	Male	Basketball	47	16
4	Female	Athletics	49	21
5	Male	Rugby	43	13
6	Female	Hockey	46	15
7	Male	Judo	42	13
8	Male	Volleyball	51	22
9	Male	Archery	48	14
10	Female	Squash	43	14
11	Female	Gymnastics	47	15
12	Male	Football	53	21

#### Data Collection

In study 1, coaches were interviewed in depth to ascertain the nature and extent of their understanding and use of reflection, together with any benefits they felt it accrued. Each interview lasted between 50 and 60 min. Questions were deliberately left open, focusing on their use, or lack thereof of RP during their coaching careers. Against the purposes of the study, one central question was ‘what impact has RP had on your coaching practice?’ Other elements asked participants to consider what factors *had* played a central role in their development as a coach? Against these purposes, the nature of the interview allowed the researcher to guide the discussion, while at the same time, enabling the participant to highlight the areas of their coaching development they felt were important.

#### Data Analysis and Credibility

Once the interviews were transcribed, an inductive thematic analysis was completed in accordance with the steps outlined by Braun and Clarke (2006). This process enabled data credibility by: (a) the independent coding of the data (i.e., investigators’ triangulation); (b) the checking of the categorisation process by three researchers experienced in qualitative methods (Lincoln and Guba, 1985); and (c) examination by the participants of the researchers’ scripts and their interpretation to ensure that the data collected was authentic and reflected their experiences (Birt et al., 2016). This practice of member reflection—the opportunity for coaches (members) to reflect on and, if appropriate, query particular aspects of the interpretation of the data they provided represents good practice in qualitative research (*cf*. Smith and McGannon, 2017). As advocated by Creswell (2009), the process was carried out with analysed data rather than transcripts. This was important to retain the diversity and produce the higher level, overarching themes (Braun and Clarke, 2006), while also offering participants the best opportunity to contribute to the picture generated.

### Results

Data from part 1 revealed three key themes arising from 122 meaning units gathered from the 12 participant coaches. These themes were as: influential people; early coaching incidents; and experiences of RP. The derivation of these higher order themes is shown in Table 2.

**Table 2:** Themes from Part 1.

High order theme	Lower order themes	Data extract
Influential People	Initial impact into coaching	*I just adored my coach when I was competing—she’s the reason I got into coaching. I thought it would be cracking to continue as a coach after I finished*.
		*I got into coaching because of my coach and I suppose that I followed…. or copied the way he coached in the beginning. He was a big influence*
	Impact on coaching approach	*My coach really impacted on how I coached, especially initially but I still keep copying some of his ways*.
Early Coaching Incidents	Recognising mistakes	*But when it came to game days, I was a disaster*
		*I guess I made a lot of mistakes but it took me a long time before I took that on board*
	Need to change	*I thought the way they played—actually no—whether they won or not reflected on me as a coach. I realised later it was me that needed to change*
		*I think when I wasn’t getting the results and training wasn’t going well, I started to think that I needed to change my modus operandi*
Experiences of RP	Coaching is about action	*I do not like to reflect, I like to do*
	Clarity of understanding	*I do not think I really know what reflection is but perhaps that needs to change in coach education*
		*I’m not sure about reflection—my coach education courses kept talking about it but no-one actually explained what it was*

#### Influential People: Still Having an Impact

All participants recollected influential people who had made a positive impact on them within their sport and whose influence had subsequently prompted them to start coaching. The female swimming coach stated as: *I just adored my coach when I was competing—she is the reason I got into coaching. I thought it would be cracking to continue as a coach after I finished*. These early motivations were easily recalled by all participant coaches. The basketball coach remembered positive experiences, saying: *Oh yes! I had a great time in sport and, you know, I was never that good. I was OK, but it was the team and especially the coach that kept me coming back*. This importance and influence continued from recruitment into coaching through the initial stages for all participants. The male swimming coach said as:

My first coaching job was while I was still at school and still swimming competitively. I did not have any qualifications or experience, but I loved it, especially as Mike was there to help. He was this older guy, way more experienced than me and had been coaching swimming for over 20 years. We did not always agree as I thought I knew best and to be fair Mike was a bit of a dinosaur, and a parent, but he really did encourage me to think about what I was doing rather than just copying him.

Developing this point, the archery coach relayed his experiences within his sporting organisation, saying:

I had never really thought about going into coaching as I was still competing, admittedly at not such a high level as I had been. A couple of people, who were involved as officials in archery, kept speaking to me at competitions, saying that they needed to keep people in the sport, especially the good ones.

#### Early Coaching Incidents: Changing Thinking

In their accounts, all the coaches could remember events that were significant to them and their subsequent development within their sport. The basketball coach remembered,

I started off running a junior basketball team—10 years old—and loved it. The kids just wanted to play, and the parents were great too—very supportive. But when it came to game days, I was a disaster. I thought the way they played—actually no—whether they won or not reflected on me as a coach. I realised later it was me that needed to change.

The tennis coach related a similar example, again relating to competition, rather than the practice sessions, saying,

When I started in coaching, I remember going to a tennis competition with a few of the players. I spent the day doing everything wrong. This player’s footwork was sloppy, the next player just did not understand the tactics and the third, well he just seemed to be so tentative and unsure. So, what did I do? I spent the whole day yelling at them so none of us had a good day. It wasn’t until a lot later that I connected the dots—no-one had reinforced the importance of footwork or taught tactics so how could I expect these kids to know. It caused me a radical rethink, I tell you. But probably too late for these players!

Both of these incidents related to coaching behaviour in competition, rather than practice, usually a more dynamic and less controlled environment. Notably, however, these incidents were still memorable as learning experiences. Importantly, both these themes showed alternative influences which were clearly more useful and important to these participant coaches.

#### Reflective Practice: The Need for Action

All coaches in these initial interviews did not view reflection in a positive light. The football coach said as: *I do not like to reflect, I like to do…that is why I got into sport!* This position was supported by the volleyball coach, who stated*: I keep hearing about how important reflection is to coaching but I have never reflected—no-one has told me how to. I just do not get it*. The rugby coach thought: *You have to overthink yourself when you reflect and I do not have the patience for that. I like to take action, sort things and make decisions—that makes things better*.

Other coaches demonstrated a lack of awareness of reflective practice; for example, the gymnastics coach said: *Do I reflect? I have no idea!* The judo coach added: *I am not sure about reflection—my coach education courses kept talking about it but no-one actually explained what it was*. The hockey coach reinforced this idea, stating *I keep hearing about reflection but not sure how this differs from evaluation*. These examples highlighted a genuine lack of understanding of RP and, at the same time, belittling of potential learning experiences.

### Discussion

There are a number of key messages arising from the Part 1 data. Notably, in addition to their own experiences, all coaches recalled when questioned that athletes and colleagues influenced them; coaches clearly recollected the importance of early encounters in their respective coaching pathways. With reference to early coaching incidents, evidence of reflective thought was made apparent, despite the coaches’ claims to the contrary. Indeed, specific memories of salient coaching episodes appeared to influence current practice and inform beliefs and remained important for years afterwards. For example, the need for action, as opposed to thought, exhibited by the rugby and football coaches, and the realisation of the tennis coach of the plethora of skills, cognitions, and behaviour required to function and perform effectively in a competitive setting clearly illustrate this: both examples highlight the need for ‘on-the-job learning’ to enable each interviewee to make sense of, and form new patterns of behaviour and thoughts as a result of these memories.

Analysis of Part 1 demonstrates that coaches cite events which hold value and inform subsequent action. While coaches are able to recall key memories and describe how they have informed their development—counter-intuitively—they see little value in learning how to capitalise upon their experiences to best effect. Paradoxically, experience is held as key, whereas RP is not regarded as necessary—despite high prestige organisations (Helyer, 2015) and certain coach education programmes advocating RP as a developmental tool, without equipping coaches with the necessary knowledge and skills to implement effectively.

Rather, what is occurring can be likened to the principles that govern andragogy, which suggest that RP is something that these coaches do spontaneously (Sato and Haegele, 2017), rather than it forming a high value learning strategy. Yet, it is clear that pertinent experiences that hold significant potential for the development of each coach’s expertise are stored in their respective episodic, long-term memories.

At present, and only to a limited extent, the recollection of coaching experiences and actions informs decision making; however, in this cohort of coaches, these memories are not routinely repurposed to intentionally enrich professional development. Yes, they have experiences that inform the way they coach in the present, but RP and the interrogation of memories to consciously change praxis and enhance expertise remains undeveloped. Participants’ recall of early coaching incidents was vivid, but they used their current knowledge as a lens to make sense of these early episodes, rather than questioning how they processed information available to them that related directly to their own dynamic environments (Mees et al., 2020). Consequently, we concluded that sub-optimum, unstructured RP was apparent, and it was not deployed as a conscious tool to enhance professional development. Therefore, given that these coaches did not consider that they used RP, and indeed placed limited value on the practice of RP, the schism between perception and action is worthy of further investigation. In summary, as the participants clearly still seek to develop their expertise, these findings present a clear basis through which to establish the fundamental potential of RP within these coaches’ repertoire of skills.

Consequently, there is a clear mandate and educational need to address gaps in this cohort’s knowledge with regard to RP. Therefore, in light of what was established in Part 1, and given the work of theorists reviewed in the introduction, we amended the direction of this research study. In part 2, we seek to structure coaches’ reflection on their coaching experience. We hope that this will elicit a cognitive, attitudinal and behavioural response in participants, as well as documenting the methodological process used in the hope that a contribution to applied research conducted on coach education can be made.

## Part 2: Educating About/Changing Perceptions of RP

Clearly, our sample showed a lack of curiosity, understanding and therefore respect for RP, despite the *post-hoc* descriptions of key people and salient teachable moments that were subsequently reported to influence practice. Accordingly, we next sought to determine if information about and guided experience in using RP would have a positive impact on these experienced coaches’ perceptions and reported efficacy of their own coaching practice.

Studies have indicated that, when coaches are influenced by the principles of RP, they are more likely to consider their coaching practice in relation to the wider context; specifically, the culture of performance, the organisation itself, support services, peers, and the performers with whom they work (Leduc et al., 2012; Peel et al., 2013; Kearney et al., 2018). Consequently, a useful means of gathering rich data in relation to coaches’ reflective practice are the use of portfolios which can be shared with peers. Research carried out by Hubbell and Robertson (2004) demonstrated that the compilation and reflection upon individual’s praxis, and the subsequent dissemination to one’s peers enabled the exchange of ideas and furthered coaches’ professional development goals. Notably, however, the majority of these studies has been carried out in the context of higher education, rather than formal coach education, where engaging with the reflective process and completion of logbooks are assessed (Nash, 2004).

In order to take advantage of opportunities throughout their career, coaches should be encouraged to become more knowledgeable about and potentially develop skills in RP. For RP to become ingrained, a key implication derived from Part 1 is that the principles and practice of RP are instilled and embedded in coach education and developmental opportunities as early as possible. However, coach developers require the requisite expertise to deliver this initiative effectively. Therefore, the question still remains as to how coaches can best acquire RP skills—unless they are involved with tertiary education.

However, there appears to be an assumption that sports coaches will develop these RP skills through immersion in the practice of coaching and *via* interactions with their peers (Nash, 2021). For many, it is construed that a certain degree of observation and mimicry exists in life and in sports coaching (Decety et al., 2002). It is assumed that RP will naturally evolve as individuals gain experience in the socially driven world of sports coaching (*cf*. Stoszkowski and Collins, 2014). This process may not, however, develop RP in an optimum manner. Such concerns notwithstanding and focusing on the social drive to emulate one’s peers, RP centred coaching practice should be situated as a natural extension to formal coach learning and development. Providing RP as an option to interested parties affords these individuals with a degree of reflexivity in the pursuit of career development and the furtherance of their expertise. Adopting RP may, especially initially, be very unsettling to practice. In this regard, Pollner (1991, p. 370) describes reflexivity as exactly that an ‘unsettling’ and is defined as ‘an insecurity regarding the basic assumptions, discourse, and practices used in describing reality’. In contrast, however, the enormity of the coaching task highlighted by these coaches, coupled with their expectations of doing rather than thinking, would appear to make reflection a luxury, rather than a valuable tool for adult learners (Hedberg, 2009). Furthermore, while the concept of ‘unsettling’ (Pollner, 1991) can be of value to the individual coaches as they seek to re-orientate themselves, reflection and reflexivity have been viewed as a threat by certain organisations (Heel et al., 2006). In short, even if the individual was tempted to try RP, their formal organisation or informal community of practice may view this development in a less than positive light. Therefore, consideration of the coaches’ milieu in which they operate and the presence or absence of like-minded practitioners is key. RP can pave the way to inculcate change, but Carver et al. (2014) state that development *only* happens in learning organisations within communities of *supportive* coaches *sympathetic to* its application.

Accordingly, these elements were built into the design of a RP intervention, planned specifically as CPD for the experienced coaches that took part in Part 1 of the present study. Specifically, we were interested in their perceptions of the process of RP and, primarily, how it impacted upon their coaching practice.

### Methods

#### Participants

All participants that featured in Part 1 agreed to participate in Part 2.

#### Procedure

The procedure for Part 2 of the study is shown in Table 3.

**Table 3:** Overview of procedure.

Process of delivery	Indicative content	Mode of analysis	Outcome
Step 1:	Reflective development workshop	‘What are you taking from this’ symposium	Transfer of knowledge into applied practice
Step 2:	Initial observation of coaching session	Observational assay	Preparation for recording of events for use in step 3
Step 3:	Initial stimulated recall	Key moments analysis	Recognising teachable moments for reflection and familiarisation with process of stimulated recall
Step 4:	Stimulated recall when observing video	Video analysis	Prompting memory and aiding accurate reflection
Step 5:	Response to interviewer questions	Accessing cognitive processes and verbalising thoughts	Reflecting to enhance expertise in coaching
Step 6:	Final review interview	Synopsis: where now?	Contemplating changes to practice

In Part 2, each coach took part in online Reflective Development Workshops (Step 1). These workshops consisted of a series of videos (3), outlining different methods of reflection, plus activities designed to elicit experiences and knowledge from their episodic memory. These experiences were then considered and, if appropriate, put into action. The videos employed reflective cycle of Gibbs (1988) and Johns (2009) model of structured reflection and Rolfe et al.’s (2001) work on RP. Although each online learning workshop was approximately 3 h in length, the practices derived from it required a minimum of 3 weeks for each coach to implement, consolidate their learning, and put the developed RP activities into practice. Therefore, in response to each reflective workshop, coaches were required to expend considerable time to engage with the learning material.

Step 3 of the data collection process involved an initial observation of each coach coaching in their favoured environment. The purpose of these initial observations was to view each coach’s pedagogical approach, determine the logistics for recording and feedback, and explain the unfolding process to each participant. This opportunity also enabled the researchers to familiarise each coach with the stimulated recall techniques (Step 4): stimulated recall (Calderhead, 1981; Messmer, 2014) is an advanced observation protocol that involves replaying video-recorded segments of a teacher’s/coach’s session and asking questions regarding their practice, decision making, and reasoning. The video footage helps to unpick what transpired during a coaching session and enables coaches to move beyond observation and evaluation to substantive reflection. Schön (1987) emphasised the role of reflection in relation to knowledge and action, namely, reflecting *on* action and reflecting *in* action. Consequently, the use of stimulated recall was employed to access metacognitive aspects of coaching and focuses on coach thinking and decision making *in* and *on* observable actions.

Coaches were invited to provide a commentary as they viewed the selected video sequences of their own performance (Step 5). In order to access cognitive processes, introspective process tracing methodology, such as stimulated recall, is most frequently utilised based on the tested assumption that a respondent can verbalise them at some level (Ericsson and Simon, 1993). Of course, coaches *have* been shown to use tacit knowledge, especially more experienced coaches, but the importance of declarative knowledge should also be highlighted and explored (Nash and Collins, 2006). To facilitate this, participants were guided through this initial session as a type of pilot study. With the use of video to stimulate recall, participants are required to explain a decision or thought process that they engage in without pre-planned thinking, rather they are responding to visual stimuli (Shubert and Meredith, 2015). These protocols have been used extensively in sport but mostly from the sporting participant’s perspective [McPherson (1999, 2000) in tennis and baseball; Whitehead et al. (2018) long distance running; Samson et al. (2017), snooker; Welsh et al. (2018), and Arsal et al. (2016), in golf], rather than with coaches. More recently stimulated recall has been used to examine the practices of sport coaches (Whitehead et al., 2016), the primary rationale being to explore the cognitive processes being employed while viewing observable actions (Eccles and Güler, 2017). In the present study, the stimulated recall interviews required the coaches to report what they were thinking and/or feeling during a previous coaching episode (Step 6). Coaches were instructed to describe their emotions and cognitions upon observing their actions and communications. They were told that they could stop the recording at any moment they deemed to be significant to explain these thoughts, feelings and any resultant actions. If required, they were also encouraged to rewind the tape to review any situations that were notable.

While watching the videos of their practice, the coaches provided a commentary upon the visual stimuli presented to them. In so doing, the coaches were limited in their capacity to comment upon the unconscious or tacit processes that underpinned their actions. To account for this shortfall, coaches were then required to respond to questions posed by the researcher (Step 7; for further information see Ericsson and Simon, 1993). These questions were designed to elicit deeper thought and meaning from each of the coaches by questioning their think-aloud deliberations and represented an important addition to the simpler think aloud, narrative that occurred in step 4.

The purpose of the questioning was varied, ranging from clarification, exploring different options and changing the frames of reference. Steps 6 and 7 in the data collection process were repeated on three separate occasions over a 6-month time period. At the end of this process, there was a final review interview (Step 8) to enable the coaches to reflect back over the entirety of the data collection (Table 3: Steps 1–7).

#### Data Analysis and Credibility

Data were collated from these coaches at two main points within the process. First, as they were participating in the stimulated recall intervention and, second, in the final review interview. These data were analysed in a similar manner to Part 1.

### Results

Findings are presented below, while the major themes are presented in Table 4.

**Table 4:** Themes from Part 2.

Step within process (see Table 3)	Timescale	Theme	Data extract
6 and 7	Stimulated recall	Engagement with the process	My initial thought was ‘I do not like this. I feel put on the spot, having to justify my actions and I found that difficult’. But actually, it really helped as I got more used to it but it is a very uncomfortable experience.
			I’ve never done this before so it is completely new—not sure it is helping.
		Questioning practice	I always thought I asked questions, you know, to see if the players understood. But now I have more questions that I ask myself, not just at training either. The players are also saying they understand better, at a deeper level, if you know what I mean?
			I have never asked myself or the players questions. I really see how this could help going forward.
		Do I Reflect?	I think there has been a sort of validation process of my thinking—I’m not sure I would have ever considered that I was right. I was always wary of trusting myself, not sure where my ideas came from.
			This is really causing me to question everything. Sometimes that’s good but now I find myself re-running the video in my head while I’m coaching. It’s making me think more but sometimes that is affecting my ability to act, to make decisions, especially quickly. I do not just react anymore.
8	Final review interview	Benefits of reflection	I wish I had understood this when I was doing my Level 1. I had to fill out a reflective journal and found it hard because I did not understand what to write—these workshops would help.
			I kept hearing how important reflection was, it really helped you develop as a coach, made you think critically and come up with other ways of doing things….I mean how important could it be if I was never shown how to do this. I’ve been a coach for a lot of years, but reflection was always this big mystery, the holy grail, now I understand but why could not I have this earlier?

#### Stimulated Recall

During the video stimulated recall phases, coaches were very engaged with the process, especially once they became accustomed to the method. During latter video-stimulated discussions, there were less questions as the coaches were generally noticing more.

##### Stimulated Recall: Engagement With the Process

The stimulated recall protocol was new to all participants, so there was a period of time when they were becoming more accustomed to explaining what they were doing, as the judo coach highlighted, saying,

Concentrating on bits of my coaching from a video was a really different experience. I have not really seen myself coach before….I mean I have had feedback on my coaching but never actually seen myself. Takes a bit of getting used to.

The coaches agreed that the process was challenging but did consider that there were benefits to be gained in the latter stages. As the football coach said,

My initial thought was ‘I do not like this. I feel put on the spot, having to justify my actions and I found that difficult’. But actually, it really helped as I got more used to it but it is a very uncomfortable experience.

The more the process continued, the more positive the coaches’ feedback became. For example, the athletics coach provided specific instances as well as resulting changes that she attributed to the process, stating

I found the talking about the video really helpful. At first, I was not really sure what to be looking at in my coaching so stopping the session did not really happen very often. The questions helped me focus on elements within the session and made me examine them more closely. I also found that I was becoming more confident and aware of what I was actually doing within my sessions.

Not all of the coaches reported specific examples. However, the co-construction of knowledge through the questions posed by the lead researcher was evident. This allowed the coaches to develop strategies for RP which was a key feature of these shared video sessions (Vygotsky, 1978).

##### Stimulated Recall: Questioning Practice

Once these coaches became more confident in the process, they started to ask more questions about their practice rather than the procedural aspects of data collection. For many coaches, seeing themselves on video was a new experience, as the rugby coach highlighted the difference between his perceptions of his coaching vs. the observed actuality, saying

So, I am looking at this and wondering why I reacted like that. It seems a bit OTT (over the top) and I did not think I would …if you asked me, I would say ‘no, I do not do that.’ But I am watching it, so I must!

This questioning of coach behaviour during practice evolved into a more general questioning of established principles and practice design. For example, the basketball coach provided some interesting perspectives around his reliance on tacit knowledge, stating,

I know in my gut when something is working or not working. That practice did not work here, and I think it was my fault. I did not do enough preparation work so that they would grasp the concepts that I was trying to put across. It just failed. I learned from that failure, but I will try it again but from a different perspective.

This questioning of practice principles was also reported to be transferred from the viewing of video into the training ground as the football coach noted; *It is interesting because I think some of the team have picked up on this. I mean Davie said that training seemed more focused, nothing major but we seemed to do more now and get more from each session*. The squash coach reinforced this, stating,

I always thought I asked questions, you know, to see if the players understood. But now I have more questions that I ask myself, not just at training either. The players are also saying they understand better, at a deeper level, if you know what I mean?

This perceived emphasis on ‘sense making’ by questioning practice, appeared to allow coaches to highlight and reflect upon aspects that could be more tightly focused, affording less reliance on established routines and more recognition of learning opportunities.

##### Stimulated Recall: Do I Reflect?

In Part 1, these coaches insisted they did not reflect as part of their practice; indeed, this was a criterion for inclusion in the study. However, these coaches were now questioning whether this was the case. The judo coach considered, *Some of the questions I am being asked are the same as I would ask myself. I think that I am now anticipating some of the questions but in reality, I probably was already asking them*. The athletics coach agreed with much needed confirmation of her coaching approach, saying, *I think there has been a sort of validation process of my thinking—I am not sure I would have ever considered that I was right. I was always wary of trusting myself, not sure where my ideas came from*.

The volleyball coach experienced apprehension at the thought of explaining his thoughts and actions but discovered the reflective process to be beneficial, thinking,

It is nice to be able to answer the questions and explain my thinking. It all seems to have crystallised with me—I was apprehensive that I would not be able to explain my actions while coaching, never mind my thoughts but actually once I understood what was needed it was actually very helpful, in a slightly intimidating way.

The coaches appeared to have realised that, whether they initially considered that they reflected or not, they were applying many of the principles of RP. The stimulated recall intervention and subsequent questioning had given them more confidence in their cognitive processes as well as more tools to use in their coaching.

#### Final Review Interview

##### Final Review: Benefits of Reflection

Reflection is said to allow coaches to consciously develop their coaching practice by becoming more self-aware through a process of self-evaluation (Anderson et al., 2004). The participant coaches were selected for this study due to their own perceived lack of RP, but it should be acknowledged that all actually employed reflective principles and practices. However, they did not find formalising this a simple or easy process; as exemplified by the volleyball coach, who thought; *I used to find it difficult to get into the zone because trivial thoughts kept intruding*. Similarly, the tennis coach considered *Reflection is my private space to get away from it all and think!* The rugby coach said, *I wish I had understood this when I was doing my Level 1. I had to fill out a reflective journal and found it hard because I did not understand what to write—these workshops would help*.

The judo coach summed up his final thoughts on the benefits of reflection for him, stating,

I found the workshops really useful—some of it was an endorsement of what I had already being doing over the years and that is always good but also, I got some new ideas. Some different ways of doing things, different approaches, new ways of looking at things. It did not require a major shift but gave me more ammunition for my practice.

There was discussion around the difficulties associated with changing long established practices, but also the benefits of new approaches. As the football coach exemplified as: *I used to do things in a certain way, my sessions were very structured and followed a pattern. I really resisted any type of change—it did not suit the way I wanted to do things*. This point of view was reinforced by the rugby coach who similarly found new approaches at odds with his existing practice, saying,

Initially I could not see why I needed to change anything, or even reflect on my practice. I thought I was successful, effective, able to adapt but really it made me realise that there were things I needed to do better. That was hard as I could not see the benefit initially.

The athletics coach was equally enthusiastic about the benefits of reflection but highlighted the lack of clarity and process around reflection in her coaching in previous years, saying,

I kept hearing how important reflection was, it really helped you develop as a coach, made you think critically and come up with other ways of doing things….I mean how important could it be if I was never shown how to do this? I’ve been a coach for a lot of years, but reflection was always this big mystery, the holy grail, now I understand but why could not I have this earlier?

One of the more unexpected findings of this study was the relationship described by these coaches between critical reflection and innovation and creativity. The coaches in this study reported increases in both abstract and conceptual thinking, coupled with enhanced problem solving directly related to their coaching context. We will investigate this further in a future paper.

## Concluding Thoughts

The aims of this research were twofold: Part 1 aimed to examine high level coaches’ understanding of their learning journey and their knowledge of reflection; Part 2 included an intervention to further these participant coaches’ knowledge and integration of reflection into their coaching practice. In the context of sport, reflection is believed to enable coaches to link their knowledge to practice, helping them to improve and develop. An early paper of Knowles et al. (2001) noted that the development of expertise is ‘often acquired through a mixture of “professional knowledge” based programmes (e.g., academic courses or coaching awards) and practical experience within the sports setting (through supervised experience or in/post-course placement)’ (p. 185). The coaches in this study were already reflective, although they did not recognise that they were in fact engaging in reflective process. Coaching can be a challenging environment and by the end of the educational intervention, aided by self-reflection and reflexivity, these coaches became more self-aware, enabling them to become better problem solvers and to improve their decision making (Saunders et al., 2006).

Returning to our previous point about what might have been missing in earlier RP work, it is important to consider the mechanism through which the intervention in Part 2 exerted its primary effect. While watching the video of their practice, coaches initially required additional prompts to enable a deep and meaningful discussion of their practice—otherwise a running commentary ensued, as opposed to an exposition of coaching practice. During the latter stages of the study, however, the participants were much less dependent on the lead researcher’s questions and prompts.

In coaching, knowledge and experience alone are insufficient to develop and thrive in such a dynamic environment. If coaching was not dynamic enough—human performance is never static, either. Consequently, coaches are required or will feel pressure to continually seek to improve. One tactic to cope with the pressure is to mimic what other coaches do. However, without the accompanying declarative knowledge, such mimicry is at best a short-term solution. On a more negative note, mimicry can be construed as an unwillingness to interrogate one’s own practices to see, if and how, other coach’s practices may or may not be relevant to their praxis. In this regard, Stoszkowski and Collins (2015) suggested that the acceptance of new information in any learning experience will be dependent on its compatibility with a coach’s existing schemas.

Coaches in the present study reported benefitting from the individual interaction and challenge resulting from the video viewing. In order for schemas to be revised the process of ‘unsettling’ mentioned earlier by Pollner (1991), highlights the often disruptive, but positive effect of the questions posed. Coach developers need to be aware that effective RP is unlikely to be formed without the input of a critical friend/coach developer to challenge, encourage and shape new thinking and learning.

Mechanistically, however, it is important to note that the unsettling effect was effective because it required coaches to probe for, expose and critically consider their actions *against their existing knowledge criteria*. That is, the coaches did not just reflect on *what* they did, but crucially, *why* they did it and *how* it could be improved. We would suggest that, without the development of these knowledge structures and decision-making processes (*cf*. PJDM—Collins et al., 2016), RP could be reduced to a bland navel gazing exercise, rumination or potentially a means of getting stuck in a cycle of negative self-talk. As an example, we would offer the very bland and surface reviews submitted by novice coaches and teachers in their logbooks and teaching practice diaries. In short, it is the combination of how to reflect *and* against what criteria that makes RP the powerful tool for behaviour change and improvement, which it has the potential to be.

This parallel content idea (how and against what) represents an important factor for the dissemination of RP in coach education schemes. These coaches highlighted their perceptions of the benefits of reflection, but strongly recommended that both coach education courses and coach developers, and ultimately sport organisations, include more information about RP rather than follow the assumption that coaches know how to reflect, and if they do not, that they will likely ‘learn on the job’.

In relation to the participants in the present study, the knowledge of what to reflect against was notably well developed. This enables them to make best use of the RP process. In part, there was less emphasis on ‘gut instinct’ from this group of participant coaches (all remember had operated at international level) at the end of study 2 in comparison to study 1. This may be due to their increased knowledge, understanding and application of reflective skills, but also because they were ‘confronted’ with the need to offer justifications for the decisions they were taking.

The ‘gut instinct’ of many coaches may be a tacit understanding that had been developed through many hours of experience and application in a coaching environment. However, in terms of developing expertise, we posit that years of coaching experience counts for a good deal—but it can also be viewed as an untapped resource if it is not utilised. If a suitable methodology is not taught and used to convert rich experiences into advanced schemas then limited self-awareness is likely to inhibit problem solving and stunt the options available with which to make effective decisions (Nash and Collins, 2006).

A potential solution is to consider a cognitive apprenticeship initially proposed by Collins et al. (1991), and later, in a coaching context by Collins et al. (2016). These authors suggest that such a cognitive apprenticeship would lead to better professional judgement and decision making by interrogating experience and using RP to add structured complexity to individual coach’s professional schematic.

## Data Availability Statement

The raw data supporting the conclusions of this article will be made available by the authors, without undue reservation.

## Ethics Statement

The studies involving human participants were reviewed and approved by Moray House School of Education, The University of Edinburgh. The patients/participants provided their written informed consent to participate in this study.

## Author Contributions

CN, AM, and DC contributed to conception and design of the study and wrote the sections of the manuscript. CN carried out the fieldwork. All authors contributed to the article and approved the submitted version.

## Conflict of Interest

DC is employed by Grey Matters Performance Ltd.

The remaining authors declare that the research was conducted in the absence of any commercial or financial relationships that could be construed as a potential conflict of interest.

## Publisher’s Note

All claims expressed in this article are solely those of the authors and do not necessarily represent those of their affiliated organizations, or those of the publisher, the editors and the reviewers. Any product that may be evaluated in this article, or claim that may be made by its manufacturer, is not guaranteed or endorsed by the publisher.
